# Prevalence of latent tuberculosis infection in healthcare workers at a hospital in Naples, Italy, a low-incidence country

**DOI:** 10.1186/s12995-016-0141-6

**Published:** 2016-11-24

**Authors:** Monica Lamberti, Mariarosaria Muoio, Antonio Arnese, Sharon Borrelli, Teresa Di Lorenzo, Elpidio Maria Garzillo, Giuseppe Signoriello, Stefania De Pascalis, Nicola Coppola, Albert Nienhaus

**Affiliations:** 1 Department of Experimental Medicine, Section of Hygiene, Occupational Medicine and Forensic Medicine, Second University of Naples, Via dei Crecchi 16, 80133 Naples, Italy; 2 Department of Mental Health and Public Medicine, Section of Infectious Diseases, Second University of Naples, Naples, Italy; 3 Institute for Health Services, Research in Dermatology and Nursing, Germany, Institution for Statutory Accident Insurance and Prevention in Healthcare and Welfare Services, University Medical Centre Hamburg-Eppendorf, Hamburg, Germany

**Keywords:** Tuberculosis, Tuberculin skin test, Healthcare workers, Quantiferon test, Health surveillance, Occupational exposure

## Abstract

**Background:**

Healthcare workers (HCWs) are at higher risk than the general population of contracting tuberculosis (TB). Moreover, although subjects with latent TB infection (LTBI) are asymptomatic and are not infectious, they may eventually develop active disease. Thus, a fundamental tool of TB control programs for HCWs is the screening and treatment of LTBI.

**Methods:**

From January 2014 to January 2015, hospital personnel at Azienda Ospedaliera Universitaria, Naples, Italy, were screened for TB. To this end, a tuberculin skin test (TST) was administered as an initial examination, unless when contraindicated, in which case the QuantiFERON® TB-Gold (QFT) assay was performed. Moreover, QFT was carried out on all TST-positive cases to confirm the initial result.

**Results:**

Of 628 personnel asked to participate, 28 (4.5%) denied consent, 533 were administered TST as the baseline examination, and 67 were tested only with QFT. In the TST group, 73 (13.2%) individuals were found positive, 418 (78.4%) were negative, and 42 (7.9%) were absent for the reading window; QFT confirmed the result in 39 (53.4%) TST-positive individuals. In the QFT-only group, 44 (65.7%) individuals were found positive. All TST- and/or QFT-positive subjects were referred for chest X-ray and examination by an infectious diseases specialist. None were found to have active TB, and were thus diagnosed with LTBI.

**Conclusions:**

Although Italy is a low-incidence country regarding TB, our findings suggest that the prevalence of LTBI in HCWs may be relatively high. As a result, active screening for TB and LTBI is needed for these workers.

**Electronic supplementary material:**

The online version of this article (doi:10.1186/s12995-016-0141-6) contains supplementary material, which is available to authorized users.

## Background

Tuberculosis (TB) is a major health problem worldwide. The World Health Organization estimates that in 2013 there were 9.0 million new cases and 1.5 million TB-related deaths [1]. Compounding this problem is multidrug-resistant TB, which globally was estimated to affect 3.5% of new TB cases in 2013. Although Italy is considered a low-incidence country for TB-the number of estimated new cases in 2013 was less than 10/100,000 inhabitants [1] – the disease is still considered a risk owing to abandonment of vaccination campaigns, wide diffusion of primary and secondary immunosuppression, and influx of immigrants [2, 3].

Compared with the general population, healthcare workers (HCWs) have a higher risk of contracting a TB infection on account of increased exposure to individuals in a contagious phase of the disease, inadequate use of personal protective equipment, and their specific working conditions, such as having to carry out activities in poorly ventilated areas [4, 5]. Median annual incidences of active TB among HCWs in countries with low, intermediate, and high rates of TB are 67, 91, and 1,180 per 100,000 persons, respectively [6].

The majority of active TB cases in HCWs occur when the risk of TB infection is underestimated and control programs are lacking. Thus, improving the understanding of TB transmission and adopting effective control measures have been recommended to reduce the risk of nosocomial infection [6, 7]. In addition, a fundamental tool for TB control programs is the screening and treatment of latent TB infection (LTBI). This is strongly recommended in many countries, including Italy. Indeed, although individuals with LTBI do not show symptoms of TB and are not infectious, about 10% are at risk of developing active disease and becoming infectious during the course of their lifetime [1]. A recent review estimated the median annual risk of LTBI among HCWs to be 2.9% in countries with a low incidence of TB and 7.2% in countries with a high incidence [6].

The main purpose of the current study was therefore to evaluate the prevalence of active TB and LTBI among hospital personnel operating in a context of low endemicity, and to assess possible associations between the outcome of the screening tests and epidemiological variables.

## Methods

From January 2014 to January 2015, a TB screening program was carried out at Azienda Ospedaliera Universitaria, Naples, Italy, a hospital with a risk classifiable as “low” according to CDC guidelines (i.e., <6TB patients/year in a setting with ≥200 in-patient beds) [8]. The Italian Society of Occupational Medicine recommends to carry out a tuberculin skin test (TST) every 6 years in healthcare structures classified as “very low” risk, every 2 years in “low” risk structures, every year if the risk is “medium”, and twice-yearly when the risk is “high” [9]. All hospital personnel, including physicians, surgeons, nurses, midwives, physiotherapists, laboratory technicians, radiographers, ambulance drivers, orderlies, and maintenance workers employed at the institution during the study period were asked to take part in this cross-sectional study. A pre-coded questionnaire on demographics, work (time in healthcare employment, type of job conducted, workplace frequented), and medical history (previous exposure to TB, TB vaccination status) was filled in by each participant.

The surveillance program required a TST as the baseline examination. The test was performed and assessed by trained personnel following standard procedures. In brief, 0.1 ml (2 TU) of the purified protein derivative RT23 (Statens Serum Institute, Copenhagen, Denmark) was injected intradermally on the volar side of the forearm of participants and read 48 to 72 h later. In accordance with national guidelines, a positive TST was defined as an induration measuring ≥10 mm [10–12].

Since vaccination data was either not available or incomplete for some participants, all TST-positive cases were then tested also with the QuantiFERON® TB-Gold (QFT) kit (Cellestis, Carnegie, Australia), a second-generation test based on an interferon-gamma release assay (IGRA) [13]. Participants with secondary immunodeficiency or a history of allergy were offered the opportunity to take the QFT test as their baseline examination in order to remove any possible risk of an allergic-type reaction [6]. Pregnant participants were also asked to take only this test because we wished to avoid multiple diagnostic exams in these woman, despite there being no evidence in the literature that adverse reactions to the Mantoux test can influence the course of pregnancy [6].

For the QFT test, 1 ml of whole blood was aliquoted into each of three QFT tubes, containing either TB-specific antigen (ESAT-6, CFP-10, and TB7.7), no antigen (negative control), or mitogen antigen (positive control), and incubated at 37 °C overnight before centrifugation, as recommended by the manufacturer’s protocol. Interferon-gamma concentration was then measured by ELISA: a reading ≥0.35 IU/ml (TB antigens minus negative control) was considered positive [14, 15].

All TST- and/or QFT-positive cases were referred for chest radiography and carefully examined by an infectious diseases specialist. We elected to suspect active TB infection in the presence of clinical symptoms such as cough, weight loss, fever, nocturnal sweating, tiredness, and/or X-ray suggestive of TB, and to confirm the disease by the presence of TB pathogens in sputum culture; a diagnosis of LTBI was given to test-positive participants not presenting with clinical or radiographic signs of active TB [2].

This cross-sectional study was performed in compliance with the Declaration of Helsinki and current healthcare standards, according to the recommendations of the Italian Ministry of Health [11]. All participants were informed by a physician on the rationale and aims, and written informed consent was obtained. According to Italian legislation concerning guidelines on observational studies, ethical approval for conducting this study was unnecessary, so we did not require formal approval by local institutional review boards [16]. Personal information regarding the enrolled subjects was protected according to Italian law [17].

Statistical analysis of the data was performed using SPSS v.17.0 software. Continuous variables are given as mean and standard deviation, and categorical variables as absolute and relative frequencies. Differences in means were evaluated by unpaired Student t-test, and the Chi-squared test was applied to categorical variables. Odds ratio (OR), with a 95% confidence interval (CI), was estimated by a logistic regression model to evaluate the presence of LTBI, with sex, age, work seniority, and type of employment as covariates. A *p*-value of <0.05 was considered statistically significant.

## Results

**Fig. 1:**
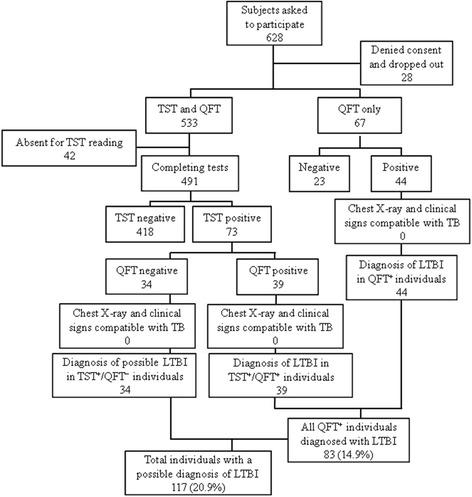
Study flow diagram and results. Abbreviations: *TST* tuberculin skin test, *QTF* QuantiFERON® TB-Gold assay

Six hundred twenty-eight personnel were asked to participate in the study: 28 (4.5%) denied consent and 42 (6.7%) failed to complete it (Fig. 1). The main demographic and epidemiological characteristics of the remaining participants are listed in Table 1: all were Italian; mean age was ~56 years; there was a slightly higher prevalence of males; mean time in employment was ~25 years; and almost half were nursing staff, with nursing and medical staff comprising over 93% of all personnel tested. No one reported knowledge of being exposed to TB outside the hospital and all were HIV-negative.

**Table 1:** Demographic and clinical characteristics of the hospital personnel completing the study

	*n* (%)^*a*^
Subjects completing the screening^*b*^	558
Age, years (mean ± SD)	56.1 ± 8
Males	324 (58.2)
Work category	
• medical staff	246 (44.1)
• nursing staff	274 (49.1)
• laboratory staff	25 (4.5)
• other^*c*^	13 (2.3)
Workplace frequented	
• medical wards	220 (39.4)
• surgical wards	171 (30.6)
• other^*d*^	167 (30)
Years in employment (mean ± SD)	24.8 **±** 9.8
TST as baseline examination	491 (88)
• positive	73 (14.8)
• negative	418 (85.2)
QTF as baseline examination	67 (12)
• positive	44 (65.7)
• negative	23 (34.3)
LTBI^*e*^	117 (20.9)
Active tuberculosis	0

*Abbreviations: TST* tuberculin skin test, *QTF* QuantiFERON® TB-Gold assay, *LTBI* latent tuberculosis infection

^*a*^values expressed as absolute frequency (percentage), unless otherwise stated

^*b*^28 subjects denied consent and 42 participants were absent during the TST reading window

^*c*^physiotherapists, orderlies, ambulance drivers, maintenance workers

^*d*^intensive care, clinical pathology, occupational medicine, audiology, radiology, microbiology

^*e*^as diagnosed for all individuals found positive at either TST or QFT

The baseline examination was the TST for 533 of the participants and the QFT assay for the remaining 67. Of the former, 73/533 (13.7%) were positive to the test, 418/533 (78.4) were negative, and 42/533 (7.9%) were absent during the reading window and were asked to repeat the test within 90 days. However, the evaluations of the retested participants occurred after January 2015, so this data was excluded from the current study. No adverse loco-regional or systemic allergic reactions were encountered. All the TST-positive subjects were then given the QFT assay: a positive result was confirmed in 39/73 (53.4%) cases.

The QFT assay was administered as the baseline examination to 52 participants with a positive history of drug allergy, to 10 pregnant women, and to 5 individuals taking immunosuppressive drugs: none reported a history of positivity to TST. Of these 67, 44 (65.7%) resulted positive and 23 (34.3%) negative.

All the 83 participants found positive with QFT (i.e., the 39 individuals positive at both tests plus the 44 individuals positive at QFT as the baseline assay) were given a chest X-ray and a careful examination by an infectious diseases specialist. Clinical and radiographic signs of active TB were excluded for all, so they were diagnosed with LTBI. In addition, although the 34 TST-positive/QFT-negative participants were strongly suspected of being falsely positive to the baseline TST (they were unable to provide documented information on TB vaccination or possible contact with non-TB mycobacteria [15]), we decided to refer them to chest X-ray and specialist examination: no evidence of TB was found in any of these subjects either.

Table 2 gives the demographic characteristics of the study population stratified for the presence of LTBI as diagnosed in all QFT-positive participants. Of these personnel with LTBI, over half were nursing staff; medical staff also accounted for a large percentage of LTBI cases, whereas laboratory technicians and other categories of workers made up only a very small fraction of this group. No statistical differences were found for sex, age, work seniority, or type of employment between the participants with and without LTBI or between those taking TST or QFT as the baseline examination. This held true also when we added the data of the 34 TST-positive/QTF-negative participants (Additional file 1: Table S1). Univariate and multivariate analysis did not identify a demographic factor associated with LTBI (Tables 2 and 3, Additional file 2: Table S2).

**Table 2:** Univariate analysis for demographic, epidemiological and occupational characteristics of the hospital personnel completing the study stratified for latent TB infection ^*a*^

	Personnel with LTBI ^*b*^	Personnel without LTBI^*b*^	*p*-value
*n*	83	475	0.47
Age, years (mean ± SD)	56.1 ± 7.2	56.1 ± 8.1	0.19
Males	45 (13.9)	279 (86.1)	0.51
Females	38 (16.2)	196 (83.8)	
Work category			
• medical staff	36 (14.6)	210 (85.4)	0.45
• nursing staff	44 (16.1)	230 (83.9)	
• laboratory staff	1 (4)	24 (96)	
• other^*c*^	2 (15.4)	11 (84.6)	
Workplace frequented			
• medical wards	33 (15)	187 (85)	0.35
• surgical wards	29 (17)	142 (83)	
• other^*d*^	21 (12.8)	146 (87.2)	
Years in employment (mean ± SD)	24.1 ± 1.0	24.9 ± 0.4	0.45

*Abbreviations: LTBI* latent tuberculosis infection, *TST* tuberculin skin test, *QTF* QuantiFERON® TB-Gold assay

^*a*^as diagnosed for TST^+^/QFT^+^ and QFT^+^-only participants

^*b*^values expressed as absolute frequency (percentage), unless otherwise stated

^*c*^physiotherapists, orderlies, ambulance drivers, maintenance workers

^*d*^intensive care, clinical pathology, occupational medicine, audiology, radiology, microbiology

**Table 3:** Multivariate analysis for demographic, epidemiological and occupational characteristics of the hospital personnel completing the study stratified for LTBI^*a*^

Variable	OR	95% CI	*p*-value
Female vs Male	1.22	0.75–2.00	0.42
Age	1.02	0.98–1.07	0.35
Years of employment	0.97	0.94–1.01	0.18
Type of employment			
• nursing vs medical	1.41	0.74–2.71	0.30
• laboratory vs medical	0.34	0.04–2.73	0.31
• other^*b*^ vs medical	1.72	0.33–8.95	0.52
Workplace			
• surgery vs medical wards	1.23	0.71–2.13	0.46
• other^*c*^ vs medical wards	0.81	0.44–1.51	0.51

*Abbreviations: LTBI* latent tuberculosis infection, *OR* odds ratio, *CI* confidence interval

^*a*^as diagnosed for TST^+^/QFT^+^ and QFT^+^-only participants

^*b*^physiotherapists, orderlies, ambulance drivers, maintenance workers

^*c*^intensive care, clinical pathology, occupational medicine, audiology, radiology, microbiology

Finally, all personnel with LTBI were referred for chemoprophylaxis. Only 6 subjects adhered to the regimen and follow-up procedure.

## Discussion

Although Italy is considered a country with a low incidence of TB [1, 13], our study identified a relatively high prevalence (20.9%) of LTBI in the personnel of one hospital, confirming that the risk of TB among HCWs is higher than that observed in the general population [7]. Indeed, a recent meta-analysis reported that, globally, the risk faced by HCWs of contracting TB is consistently higher than of the general population, indicating TB as an occupational disease [7].

In the present study, no case of active TB was encountered. In other reports on HCWs conducted in Italy and other low-incidence countries, the prevalences of TB and LTBI varied depending on the country and the type of population sample studied. For example, Franchi and colleagues evaluated 1,755 HCWs in Italy in 2004, diagnosing LTBI in 6% of cases [18]; in a study conducted in France (a low-incidence country with 5.2 cases of TB per 100,000 inhabitants in 2006), 18.9% of 148 HCWs had a positive IGRA result [19]: and in a study on 134 HCWs in Spain (another low-incidence country), the pevalence of LTBI was 11.2% [20]. By contrast, a study on 2,884 hospital workers in Portugal (a high-incidence country, with 32 cases/100,000/year) reported a very high prevalence of LTBI (i.e., 29.5%) [21]. However, a 2005 meta-analysis reported that the prevalence of LTBI in HCWs ranged 5–55% in high-income countries [22].

The 20.9% prevalence of LTBI observed in our study, which represents an intermediate value compared with previous studies, can be explained, at least in part, by our HCW population’s relatively high mean age and number of years in work. Indeed, the prevalences of LTBI and TB in HCWs are also dependent on the time that the workers are potentially exposed to the pathogen and, thus, on those two variables. For example, in a study on 2,028 HCWs in Germany (a low-incidence country), the prevalence of LTBI increased with the duration of employment, going from 5.4% in the subgroup with less than 5 years of employment, to 12.7% in those with more than 20 years in the healthcare sector; moreover, age was found to be the most important risk factor linked to a positive IGRA result (>55 years; OR: 14.7; 95% CI: 5.1–42.1) [23]. Both mean age and years of employment of the personnel enrolled in the present study (56.1 ± 8 and 24.8 ± 9.8 years) were higher than in the HCWs enrolled by Franchi and colleagues (39 ± 9 and 13 ± 8 years), who reported a much lower prevalence of LTBI [18]. The association between age and the prevalence of LTBI is also confirmed by data collected on healthcare students. In our recent study on undergraduate and postgraduate healthcare students in Italy (mean age: 25.8 ± 5.3 years), LTBI was diagnosed in only 35 of the 3,331 individuals tested (i.e., 1.05%) [24]. Comparable findings were reported by Durando and colleagues, who found a very low prevalence (0.5%) of LTBI in 881 Italian undergraduate students (mean age: 23.6 ± 3.1 years) [25].

Apart from age and years of employment, other factors that have been identified as associated with LTBI in HCWs are male gender, being born in a high-incidence country, prior exposure to TB, and a previous positive TST result. For example, Franchi and colleagues found that TST reactivity correlated with age (≥47 years) and the male gender [18]; Durando and colleagues reported an association with being born in a country with a high incidence of TB [25]; and a study on German HCWs identified associations with being foreign-born (OR: 1.99; 95% CI: 1.4–2.8), having TB in the individual’s own history (OR: 4.96; 95% CI: 1.99–12.3), and a having a previous positive TST result (OR: 3.5; 95% CI: 2.4–4.98) [23]. However, in the present study on Italian-born HCWs of relatively high age and years of employment, we did not identify any factor associated with LTBI.

The limitations of this study are principally related to the cross-sectional design used, the lack of a control group and the relatively low number of subjects evaluated. Moreover, a single testing procedure was used, with a second test (namely an IGRA) systematically carried out only in the event of a positive TST or in subjects that did not want a skin test. In our experience, TST is safe and can be used widely (we found no instance of loco-regional or systemic allergic reactions); our decision to use IGRA as a second-level examination only in subjects found positive upon TST was aimed at optimizing costs and increasing the level of diagnostic accuracy [26]. However, both tests have their drawbacks: TST has technical limitations in that it can produce results that are hard to interpret and tends to generate a significant number of false positives [27–30]; IGRA is more specific and has a sensitivity that is at least identical to that of TST [20, 31, 32], but it is more expensive and is difficult to assess around the cut-off value. Although, the 34 TST-positive but IGRA-negative subjects in our study would normally be considered as having been infected by *M. tuberculosis*, we strongly suspected they represented false positives caused, for example, by contact with non-TB mycobacteria [15, 33]; indeed, these individuals were not able to provide us with documented data on TB vaccination. We nevertheless consider it correct to use TST as a baseline exam, especially in a country like Italy where the prevalence of BCG vaccination is low [34, 35].

## Conclusions

Although Italy is considered a low-incidence country for TB, our data suggest that the prevalence of LTBI in HCWs may be high, especially in older healthcare personnel with a longer history of employment in the sector. In this epidemiological context, active screening for TB and LTBI is advised.

## Additional files

Additional file 1: Table S1. Demographic, epidemiological and clinical characteristics of the hospital personnel completing the study stratified for latent TB infection, as diagnosed in all participants found positive at TST. (DOC 41 kb)
Additional file 2: Table S2. Logistic regression analysis for independent predictors of LTBI, as diagnosed in all participants found positive at TST. (DOCX 17 kb)

## Abbreviations

HCWs: Healthcare workers
IGRA: Interferon-gamma release assay
LTBI: Latent tuberculosis infection
QFT: QuantiFERON® TB-Gold
TB: Tuberculosis
TST: Tuberculin skin test
