# Supplementary material for: Prevalence of latent tuberculosis infection in healthcare workers at a hospital in Naples, Italy, a low-incidence country
Source: J Occup Med Toxicol. 2016 Nov 24;11:53. doi: 10.1186/s12995-016-0141-6 (PMC5122022; doi:10.1186/s12995-016-0141-6)
Supplement: Additional file 1: Table S1. — Demographic, epidemiological and clinical characteristics of the hospital personnel completing the study stratified for latent TB infection, as diagnosed in all participants found positive at TST. (DOC 41 kb) [file 12995_2016_141_MOESM1_ESM.doc]

**Additional Table 1** Demographic, epidemiological and clinical characteristics of the hospital personnel completing the study stratified for latent TB infection *^a^*

|  | Personnel with LTBI*^b^* | Personnel without LTBI*^b^* | *p*-value |
| --- | --- | --- | --- |
| *n* | 117 | 441 |  |
| Age, years (mean ±SD) | 56±7 | 56±8 | 1 |
| Males  Females | 66 (20.4)  51 (21.8) | 258 (79.6)  183 (78.2) | 0.69 |
| Work category   - medical staff - nursing staff - laboratory staff - other*^c^* | 45 (18.3)  53 (22.6)  4 (16.0)  15 (28.9) | 201 (81.7)  182 (77.4)  21 (84.0)  37 (71.1) | 0.22* |
| Workplace   - medical wards - surgical wards - other*^d^* | 51 (23.2)  39 (22.8)  27 (16.2) | 169 (76.8)  132 (77.2)  140 (83.8) | 0.08** |
| Years in employment (mean ±SD) | 25±10 | 25±9.9 | 1 |

*Abbreviations: LTBI* latent tuberculosis infection, *TST* tuberculin skin test, *QTF* QuantiFERON® TB-Gold assay

*^a^*as diagnosed for all participants found positive at TST

*^b^*values expressed as absolute frequency (percentage), unless otherwise stated

*^c^*physiotherapists, orderlies, ambulance drivers, maintenance workers

*^d^*intensive care, clinical pathology, occupational medicine, audiology, radiology, microbiology

*medical staff vs. nursing staff

**medical plus surgical wards vs. other
