# Supplementary material for: Prevalence of latent tuberculosis infection in healthcare workers at a hospital in Naples, Italy, a low-incidence country
Source: J Occup Med Toxicol. 2016 Nov 24;11:53. doi: 10.1186/s12995-016-0141-6 (PMC5122022; doi:10.1186/s12995-016-0141-6)
Supplement: Additional file 2: Table S2. — Logistic regression analysis for independent predictors of LTBI, as diagnosed in all participants found positive at TST. (DOCX 17 kb) [file 12995_2016_141_MOESM2_ESM.docx]

**Additional Table 2** Logistic regression analysis for independent predictors of LTBI*^a^*

| Variable | OR | 95% CI | *p*-value |
| --- | --- | --- | --- |
| Male vs female | 1.05 | 0.68 –1.63 | 0.83 |
| Age | 1.01 | 0.98–1.07 | 0.68 |
| Years of employment | 0.98 | 0.95–1.02 | 0.47 |
| Type of employment |  |  |  |
| - nursing vs medical | 1.41 | 0.75–2.65 | 0.29 |
| - laboratory vs medical | 1.18 | 0.35–3.97 | 0.78 |
| - other*^b^* vs medical | 2.04 | 0.97–4.29 | 0.60 |
| Workplace |  |  |  |
| - surgery vs medical wards | 0.97 | 0.59–1.58 | 0.90 |
| - other*^c^* vs medical wards | 0.64 | 0.38–1.09 | 0.10 |

*Abbreviations: TST* tuberculin skin test, *QTF* QuantiFERON® TB-Gold assay, *OR* odds ratio, *CI* confidence interval

*^a^*as diagnosed for all participants found positive at TST

*^b^*physiotherapists, orderlies, ambulance drivers, maintenance workers

*^c^*intensive care, clinical pathology, occupational medicine, audiology, radiology, microbiology
